# Supplementary material for: Synergistic Effect of Conditioned Medium from Amniotic Membrane Mesenchymal Stromal Cells Combined with Paclitaxel on Ovarian Cancer Cell Viability and Migration in 2D and 3D In Vitro Models
Source: Pharmaceutics. 2025 Mar 26;17(4):420. doi: 10.3390/pharmaceutics17040420 (PMC12030038; doi:10.3390/pharmaceutics17040420)
Supplement: Supplementary file 1 [file pharmaceutics-17-00420-s001.zip › pharmaceutics-3468865-supplementary.pdf]

pharmaceutics

MDPI

Supplementary Materials: Synergistic Effect of Conditioned Medium from Amniotic Membrane Mesenchymal Stromal Cells Combined with Paclitaxel on Ovarian Cancer Cell Viability and Migration in 2D and 3D In Vitro Models

Paola Chiodelli, Patrizia Bonassi Signoroni, Elisa Scalvini, Serafina Farigu, Elisabetta Giuzzi, Alice Paini, Andrea Papait, Francesca Romana Stefani, Antonietta Rosa Silini and Ornella Parolini

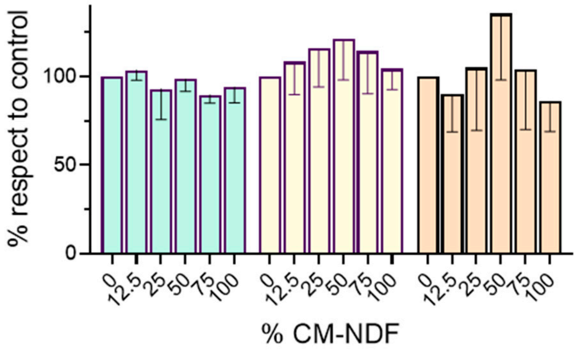

Figure S1. Effect of CM from normal dermal fibroblasts on ovarian cancer cell proliferation. HEY (green), SKOV3 (yellow) and OV-90 (orange) were treated with increasing concentration of CM collected from normal dermal fibroblasts (CM-NDF) for 48 h. Then, cell viability was assessed by MTT assay. n = 23.

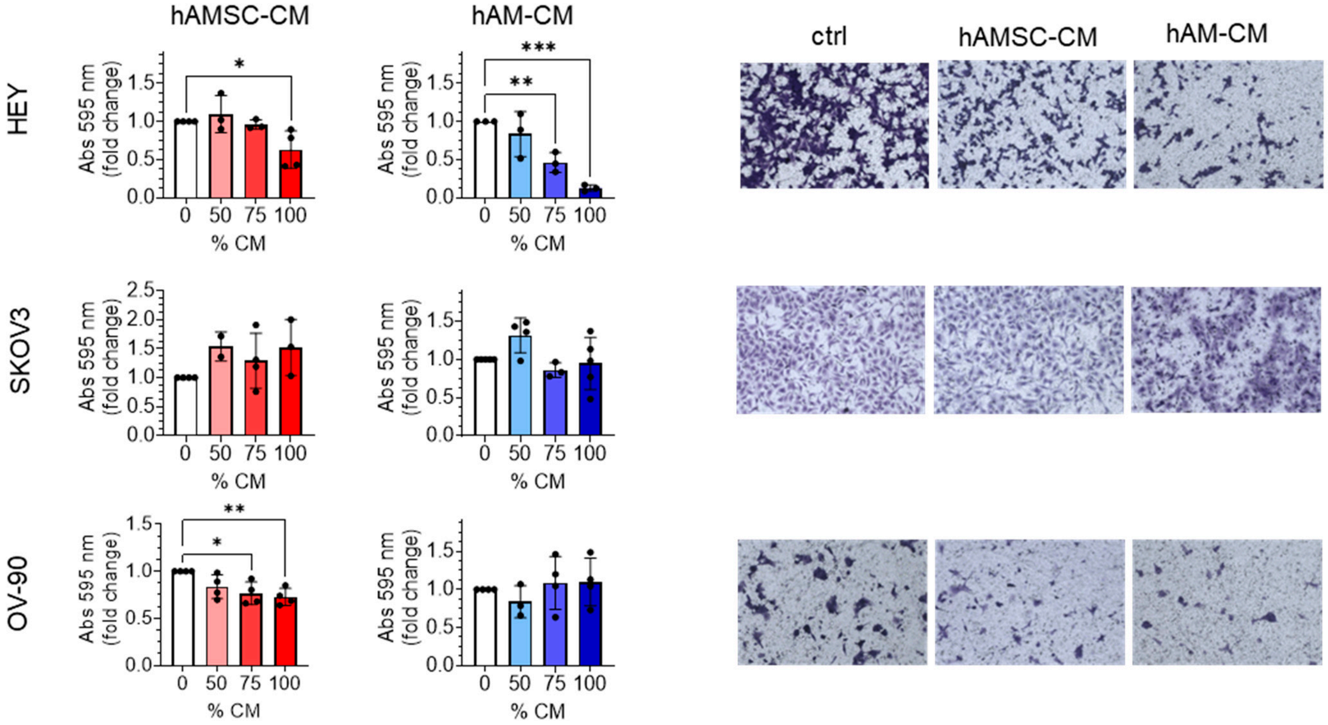

 https://doi.org/10.3390/xxxxx

Figure S2. Effect of hAMSC-CM and hAM-CM on ovarian cancer cell migration in transwell. HEY, SKOV3 and OV-90 cells were treated with 50%, 75%, or 100% hAMSC-CM or hAM-CM for 24 h. Subsequently, the cells were seeded onto transwells and incubated for an additional 24 h. Migrated cells were fixed, stained with crystal violet, and detected on the bottom side of the transwells. Migration was quantified by measuring absorbance at 595 nm following cell solubilization, and results were expressed as a fold change relative to the control. $n = 3$. * $p < 0.05$, ** $p < 0.01$, *** $p < 0.001$.

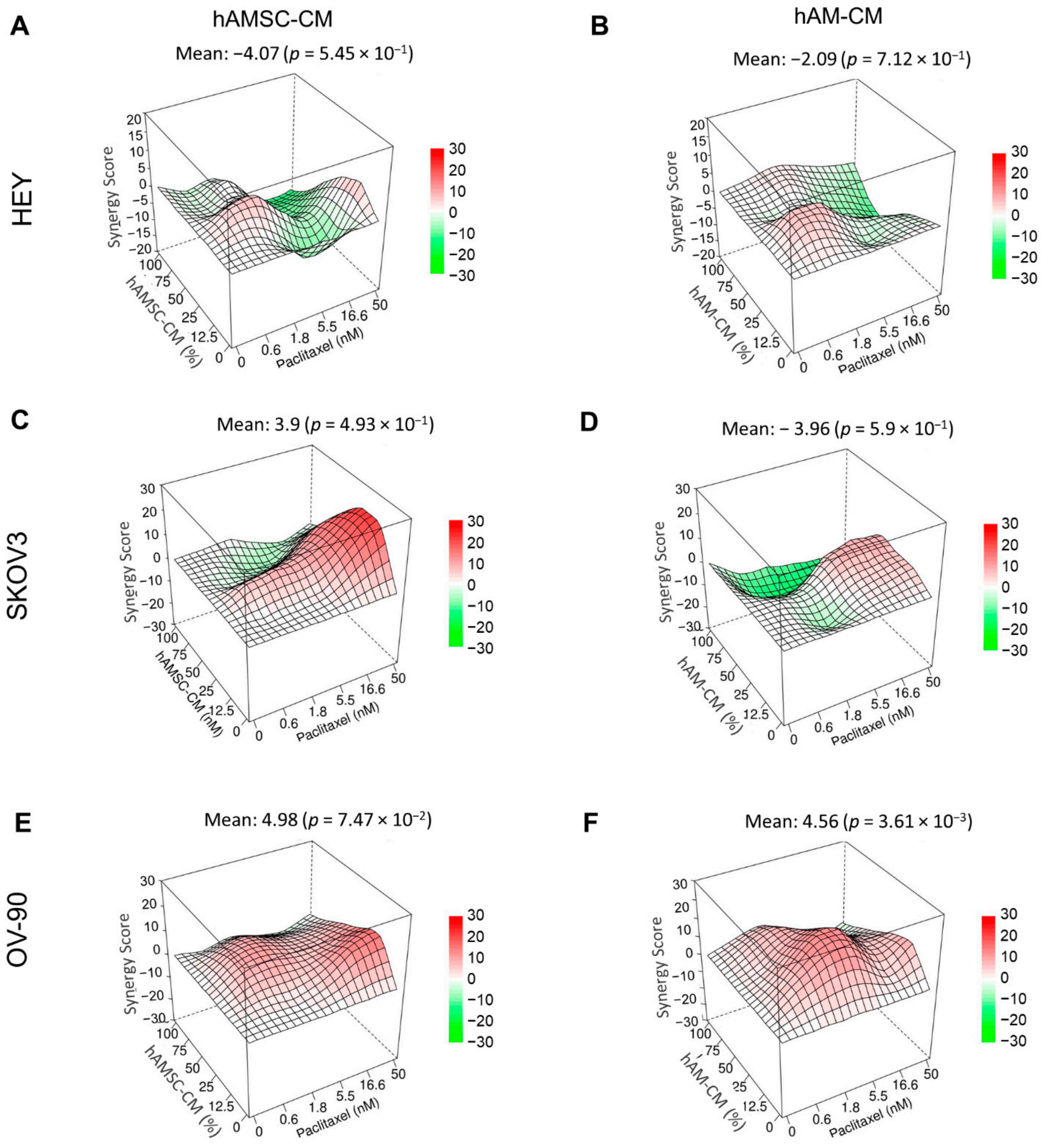

Figure S3. Synergistic effects of CM and paclitaxel on ovarian cancer cell lines in 2D. HEY (**A**, **B**), SKOV3 (**C**, **D**), and OV-90 (**E**, **F**) cells were treated with different concentrations of CM (12.5%, 25%, 50%, 75%, 100%) and paclitaxel (0.6, 1.8, 5.5, 16.6, 50 nM) for 48 h. Cell viability was assessed using the MTT assay. Results are displayed as ZIP synergy maps and synergy scores for each combination and cell line are shown.

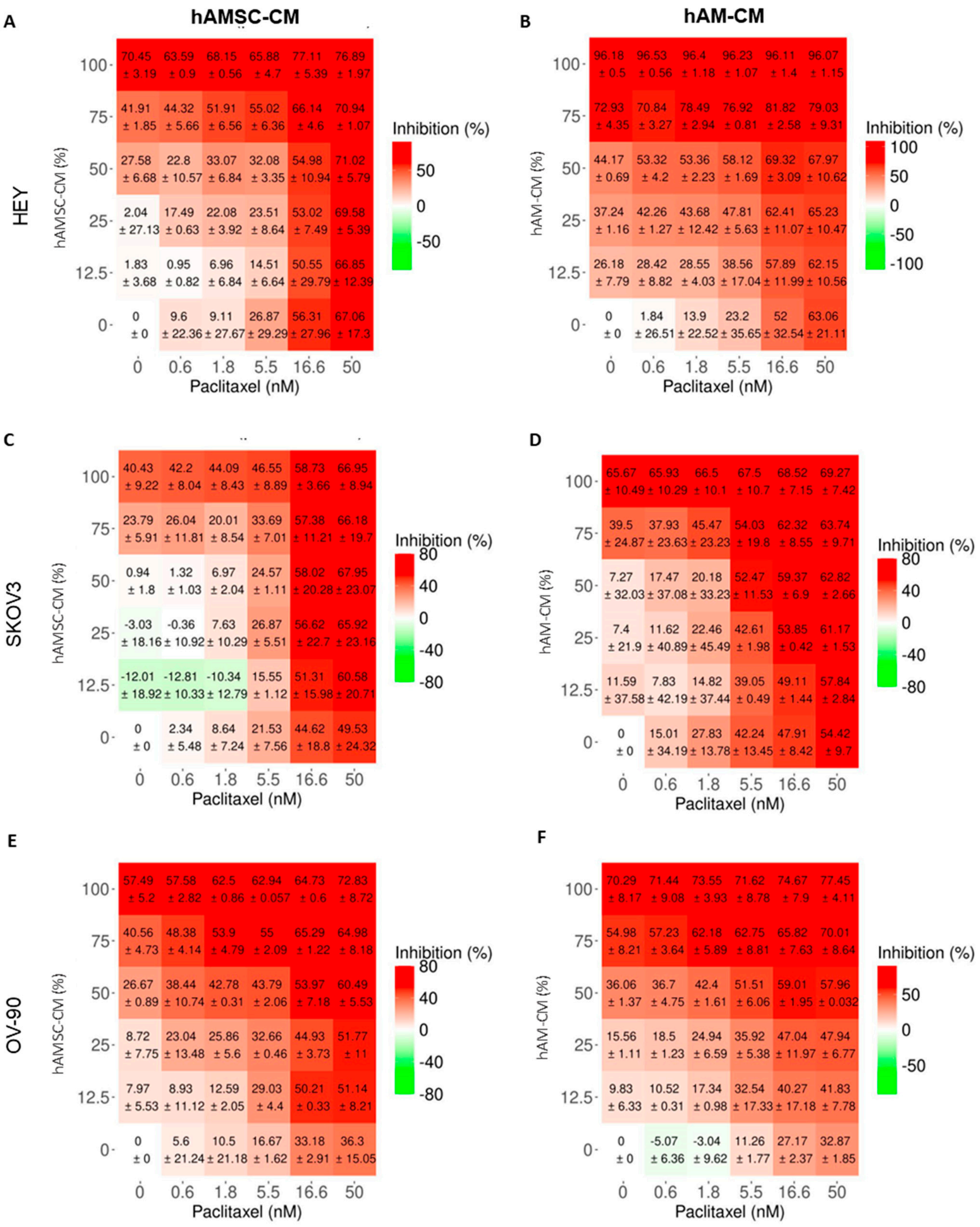

Figure S4. Synergistic effects of CM and paclitaxel on ovarian cancer cell lines in 2D- Dose response matrix**.** HEY (**A**, **B**), SKOV3 (**C**, **D**), and OV-90 (**E**, **F**) cells were treated with different concentrations of CM (12.5%, 25%, 50%, 75%, 100%) and paclitaxel (0.6, 1.8, 5.5, 16.6, 50 nM) for 48 h. Cell viability was assessed using the MTT assay. Results are displayed as dose-response matrices showing the percentage of cell viability relative to controls ± SD.

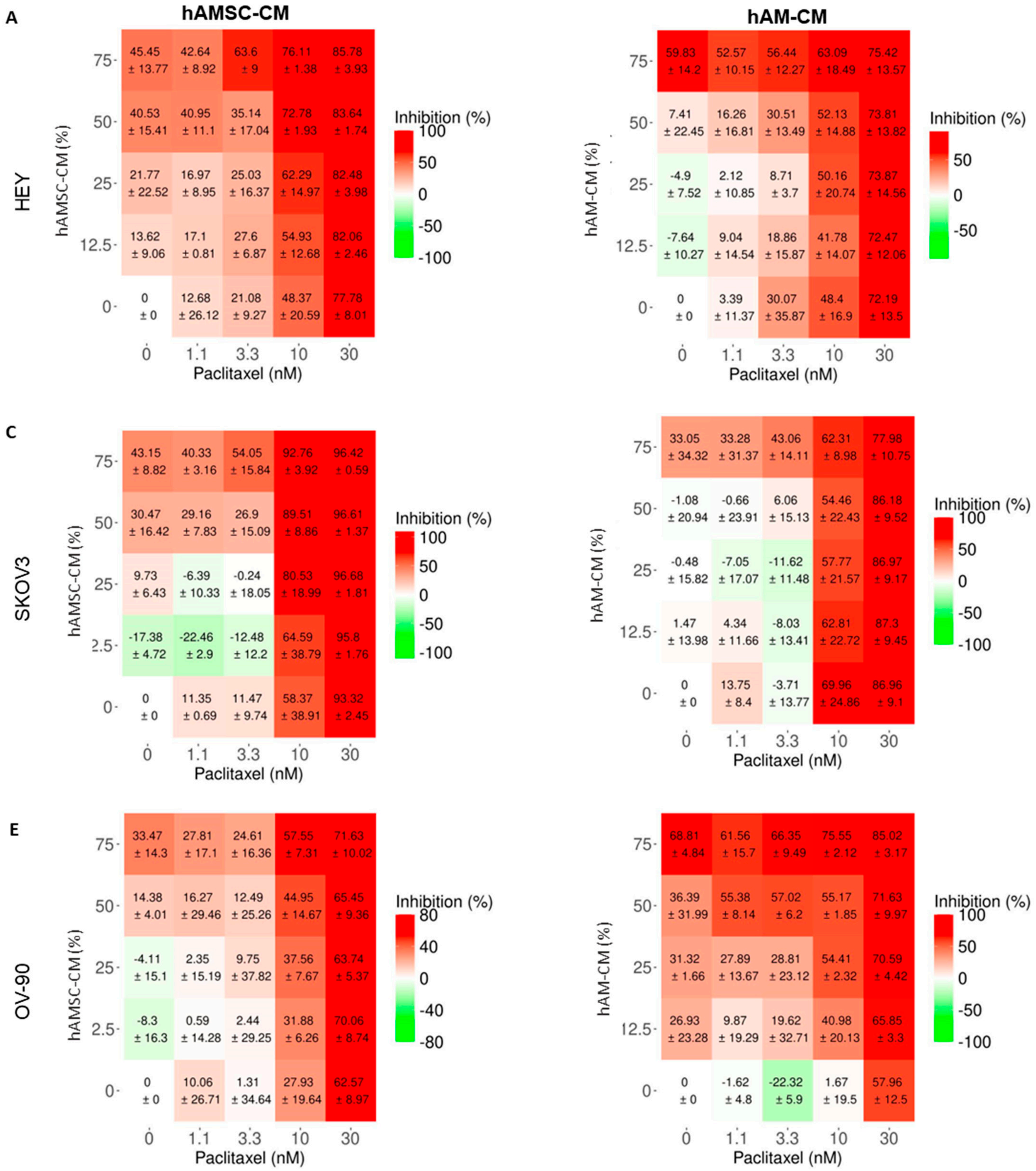

Figure S5. Synergistic effects of CM and paclitaxel on ovarian cancer spheroids in 3D-Dose response matrix. Spheroids from HEY (**A**, **B**), SKOV3 (**C**, **D**), and OV-90 (**E**, **F**) cells were treated with different concentrations of CM (12.5%, 25%, 50%, 75%) and paclitaxel (1.1, 3.3, 10, 30 nM) for 48 h. Viability was assessed using the ATP-lite assay. Results are displayed as dose-response matrices showing the percentage of viability relative to controls ± SD.
